# Contour Fillings: Their Place in Dental History

**Published:** 1891-05

**Authors:** 


### Editorial.

### CONTOUR FILLINGS: THEIR PLACE IN DENTAL HISTORY.

The extraordinary efforts recently made in several directions to manipulate history, and have it appear that the dental profession has been indebted to two men for this form of filling, seems to require a more extended notice than has been accorded it.

The controversy has in some quarters assumed an unwarranted acrimonious character, and this personal feeling has in a measure obscured the facts. The development of the operation of stopping teeth, as all readers are aware, has not been the work of any one man. It has been a gradual evolution from the simple crowding of gold into a given cavity to the adoption of perfected mechanical adjustments for the accomplishment of the same end.

The earlier period, which may be termed, to use Dr. George S. Allan's expressive phrase, the "face filling" epoch, continued up to about 1847. As far as the writer is aware, from reference and personal observation, very few attempts were made prior to this to extend the proximal fillings beyond the walls of the cavities.

About this period the active minds of Westcott, Townsend, Arthur, Dwinelle, Rich, and others were busy with efforts to produce a filling that should assume more nearly the original form of the tooth. The effort to build gold, piece by piece, lamina by lamina, was then in process of development; but the prevailing idea that all gold must be absolutely "soft" was a bar to progress in that direction.

The controversy that held the interest of the profession for a long time as to who deserved the credit for the introduction of cohesive gold was eventually very properly decided in favor of Dr. Arthur; but, in addition, it was also demonstrated that others had been making use of this form of foil some years anterior to its introduction by him.

It was well known to students of Dr. Elisha Townsend that he had a plan of "warming his gold" before filling; but the value of this was not then appreciated by those who admired his work. It is in the memory of the writer that Dr. Townsend frequently exhibited gold built up on coin as an evidence of what could be accomplished in restoring form.

That Dr. Westcott made use of the cohesive form of gold long anterior to its general adoption is quite certain; but whether he understood its real value in connection with stoppings may be questioned. The writer never had the pleasure of examining any of his work, but during a residence in Western New York, in 1847, he became familiar with the operations of one of Dr. Westcott's pupils. While at that time incapable of criticising, the fact remained that teeth were contoured with gold built out to the original form in a way not possible for ordinary methods at that period. Years subsequently, many of these very perfect fillings came again under observation, and they fully confirmed the earlier judgment. These fillings were inserted by the *aid of the mallet.*

That contour filling had its rise about this period there can be no question. It was a natural development growing out of changed conditions both in the material used and in the feeling that something better should be secured. This view is maintained by Dr. Jack ("System of Dentistry," vol. ii.), wherein he says, "The date when and by whom attention was first called to the prophylactic value of the truly contoured filling has not been clearly determined; the mode of practice appears to have been a gradual development which had grown out of the failure of the interstitial self-cleansing theories." It is impossible, however, to agree with the next paragraph, where he gives Dr. Varney the "credit not only for the most artistically produced expression in gold of the natural forms of the teeth, but of having a clear conception of their prophylactic value."

The formal introduction of cohesive gold in 1855 by Arthur led at once to the forming of gold surfaces more in accordance with the original shape. Contour fillings were common, indeed may be said to have been adopted among the better class of operators as early as 1858. Lost parts were replaced by gold built up to original and natural lines; incisors broken were reformed; entire teeth were built out with gold; molars were brought to the original masticating surfaces; in fact, all the work of the present was done thirty years ago as skilfully as it is performed to-day.

The place in dental history, then, of the work of Varney and Webb, so often quoted, is not as originators of contour filling or even of new processes, but as holding exalted positions as mechanicians, worthy always of our remembrance and regard.

The time has arrived when dentistry should take a broader view than to suppose that the man who has succeeded in placing one piece of gold upon another is the ideal dentist. Dentistry means more than this. It means a liberal culture, a quite thorough knowledge of pathological conditions. It means the philosophy of treatment, and, consequently, it means, further, a capacity for diagnosis and prognosis. It comprises more of therapeutics to-day than at any former period, and he who is unfamiliar with all collateral subjects that enter into the training of an educated dentist, with a clear conception of the possibilities before him, has no right to regard himself a worthy follower of an honorable profession.

